# Gut Metabolite Trimethylamine N-Oxide Protects INS-1 β-Cell and Rat Islet Function under Diabetic Glucolipotoxic Conditions

**DOI:** 10.3390/biom11121892

**Published:** 2021-12-17

**Authors:** Emily S. Krueger, Joseph L. Beales, Kacie B. Russon, Weston S. Elison, Jordan R. Davis, Jackson M. Hansen, Andrew P. Neilson, Jason M. Hansen, Jeffery S. Tessem

**Affiliations:** 1 Department of Nutrition, Dietetics, and Food Science, Brigham Young University, Provo, UT 84602, USA; emilys.krueger@gmail.com (E.S.K.); jlbeales@gmail.com (J.L.B.); kacie.russ@gmail.com (K.B.R.); weston.elison@gmail.com (W.S.E.); jordanricharddavis@gmail.com (J.R.D.); 2 Department of Cell Biology and Physiology, Brigham Young University, Provo, UT 84602, USA; Jacksonmhansen8@gmail.com (J.M.H.); jason_hansen@byu.edu (J.M.H.); 3 Plants for Human Health Institute, Department of Food, Bioprocessing and Nutrition Sciences, North Carolina State University, Kannapolis, NC 28081, USA; aneilso@ncsu.edu

**Keywords:** beta cell, islet, glucolipotoxicity (GLT), glucose stimulated insulin secretion (GSIS), unfolded protein response (UPR), type 2 diabetes (T2D)

## Abstract

Serum accumulation of the gut microbial metabolite trimethylamine N-oxide (TMAO) is associated with high caloric intake and type 2 diabetes (T2D). Impaired pancreatic β-cell function is a hallmark of diet-induced T2D, which is linked to hyperglycemia and hyperlipidemia. While TMAO production via the gut microbiome-liver axis is well defined, its molecular effects on metabolic tissues are unclear, since studies in various tissues show deleterious and beneficial TMAO effects. We investigated the molecular effects of TMAO on functional β-cell mass. We hypothesized that TMAO may damage functional β-cell mass by inhibiting β-cell viability, survival, proliferation, or function to promote T2D pathogenesis. We treated INS-1 832/13 β-cells and primary rat islets with physiological TMAO concentrations and compared functional β-cell mass under healthy standard cell culture (SCC) and T2D-like glucolipotoxic (GLT) conditions. GLT significantly impeded β-cell mass and function by inducing oxidative and endoplasmic reticulum (ER) stress. TMAO normalized GLT-mediated damage in β-cells and primary islet function. Acute 40µM TMAO recovered insulin production, insulin granule formation, and insulin secretion by upregulating the IRE1α unfolded protein response to GLT-induced ER and oxidative stress. These novel results demonstrate that TMAO protects β-cell function and suggest that TMAO may play a beneficial molecular role in diet-induced T2D conditions.

## 1. Introduction

High caloric intake from a Western diet rich in animal proteins and fats is linked to chronic cardiovascular and metabolic diseases, including cardiovascular disease (CVD) and type 2 diabetes (T2D), which drive global mortality and morbidity rates [1,2,3,4,5,6,7]. This diet can include high levels of quaternary amine-containing semi-essential nutrients, such as choline and carnitine, and the concomitant gut microbial metabolites are associated with pathogenic mechanisms [8,9,10,11]. Dietary excess of these nutrients is metabolized by the gut microbiome to trimethylamine prior to absorption [12,13,14,15]. At the liver, trimethylamine is oxidized by flavin-containing monooxygenase 3 (FMO3) to trimethylamine N-oxide (TMAO), which travels through the circulation and is eliminated in the urine. TMAO produced by this gut microbiome-liver axis can accumulate in the circulation and is a known chronic disease biomarker and proposed aggravator of CVD [9,13,16,17,18].

Metabolomic studies originally linked elevated serum TMAO levels to CVD through atherosclerotic mechanisms [8,19,20,21,22]. Although elevated TMAO has since been associated with other diet-mediated metabolic diseases, the direct molecular TMAO effects in the relevant tissues are unclear [6,21,23,24,25,26,27,28]. Some studies report that 7 μM to 1 M concentrations of TMAO may beneficially reduce cellular stress [9,23,29,30,31,32,33,34,35]. However, in obese T2D patients, elevated TMAO is associated with hyperglycemia, hyperlipidemia, and insulin resistance [4,5,6,7].

Within the pancreas, islets of Langerhans contain metabolic hormone-secreting cells, including β-cells, which secret insulin to manage blood glucose levels [36,37,38]. Healthy β-cell function is characterized by the proper sensing of elevated blood glucose via GLUT2, a low-affinity glucose transporter, and glucokinase [39,40,41]. The resulting signals from complete glucose metabolism through glycolysis, the tricarboxylic acid (TCA) cycle, and oxidative phosphorylation induce insulin production and secretion [41]. β-cells are highly susceptible to metabolic cellular stresses, which contribute to the T2D condition [42,43,44]. In patients consuming a Western diet, hyperglycemia and hyperlipidemia reduce functional β-cell mass to drive T2D progression [41,45,46,47,48,49,50,51,52,53]. Therefore, to elucidate the role of TMAO in T2D pathogenesis, understanding its effects on functional β-cell mass is imperative [54]. This study is the first to culture INS-1 832/13 β-cells and primary rat islets with physiological TMAO concentrations under healthy standard cell culture (SCC) or T2D-like glucolipotoxic (GLT) conditions. Since TMAO is more commonly proposed as an aggravator of chronic diseases, we hypothesized that it would reduce functional β-cell mass under SCC and worsen the T2D phenotype under GLT conditions.

## 2. Materials and Methods

### 2.1. Cell and Tissue Culture

The rat insulinoma INS-1 832/13 β-cell line was maintained in standard culture media containing RPMI 1640 + l-glutamine (Corning, Corning, NY, USA, 50-020-PB) combined with 10% fetal bovine serum (MilliporeSigma, Burlington, MA, USA, C948C69), 50ug/mL streptomycin and 50 U/mL penicillin (LONZA, Basel, Switzerland, 17-745E), 10mM HEPES (ThermoFisher, Waltham, MA, USA, 15630080), and INS-1 832/13 supplement, as described previously [55,56]. Passage doublings under 100 were seeded to culture plates (VWR, Radnor, PA, USA, 10861-698, 10861-700, 10861-702, 10861-666) (biological triplicates) at approximately 105,000 cells/mL or 40,000 cells/cm^2^ and acclimated to standard incubation overnight prior to treatment for experimental assays.

Adult wildtype Wistar rats (Charles River, Wilmington, MA, USA) were maintained under standard housing, feeding (LabDiet, St. Louis, MO, USA, Rodent 5001), and husbandry protocols. Four 3-month-old females (biological replicates) were euthanized by CO_2_, and primary islets were isolated by collagenase digestion and handpicked into standard media as described previously [57,58,59]. After 2 h of acclimation, groups of 35 islets from each animal were randomly handpicked into TMAO treatments in SCC or GLT cultures for 24 h. All animal work was approved by the Brigham Young University (BYU) Animal Committee and complied with the National Research Council’s Guidelines for the Care and Use of Laboratory Animals (IACUC Protocol Number 19-1002) and the ARRIVE guidelines. Protocol details are available as needed and upon request.

### 2.2. Experimental Culture Treatments

For SCC studies, standard culture medium was replaced with medium containing TMAO (MilliporeSigma, Burlington, MA, USA, 1184-78-7). For GLT studies, TMAO treatments were combined in GLT media containing three additional components, including 25 mM glucose (MilliporeSigma, 50-997), 0.5mM palmitate (TCI, 408-35-5), and bovine serum albumen (0.67% final concentration) (ThomasScientific, C974Z33). To incorporate the palmitate in the aqueous environment of the medium, we dissolved it in 1:1 ethanol and water and conjugated it to the albumen as described previously [60,61]. Such GLT cultures were established to model diet-driven T2D pathogenesis [51,52,62,63,64,65,66]. TMAO was dissolved in water and diluted serially for total culture concentrations between 0.3 and 160 µM, which reflects clinical levels [16,20,67,68,69,70,71,72,73,74]. This range of clinical serum TAMO levels is dependent on the method of analysis, the subject’s diet, and the disease state. T2D, Metabolic Syndrome, and CVD are predicted by serum TMAO levels over 7 or 8 µM [27,75,76,77,78]. At the most extreme, serum TMAO can increase to 170 µM in patients with various chronic diseases [5,25,68,70,72,74,75,79,80,81,82,83,84,85,86]. T2D or CVD patients present levels at or above 20 µM [5,25,68,70,72,74,75,79,80,81,82,83,84,85,86]. Alternatively, healthy patients present low levels from 1 to 3 µM [8,16,67,87,88,89]. Therefore, to represent these variable clinical TMAO concentrations, we investigated the 0.3 and 160 µM range of TMAO in both the SCC and GLT conditions. Since TMAO accumulates after a single meal containing precursor nutrients and remains elevated for the duration of the diet [13,16,87,90,91], our proof of concept studies used 24 h treatments (Figure S1A,B). Although we investigated the independent β-cell function and proliferation effects of the individual components of the GLT model, we found that the combined mixture of all the GLT components was most sufficient to model the T2D β-cell phenotype. In both the SCC and GLT experiments, no TMAO treatment (NT) controls were used as references on each of the 3 experimental cultures (biological triplicates).

### 2.3. Tetrazolium Salt MTT Viability Assay

After experimental culture, treatment media was replaced with media containing 3-(4,5-dimethylthiazol-2-yl)-2,5-diphenyl tetrazolium bromide (MTT) (CHEMIMPEX, 00697) dissolved in phosphate-buffered saline (PBS) and incubated for 4 h, as described previously [92]. Healthy mitochondrial NADH dependent dehydrogenase or superoxide dismutase reduced MTT to form tetrazolium salts and indicate cell viability [62,92,93,94]. Tetrazolium salts formed by viable cells were solubilized with acid-isopropanol, and absorbance was measured with the BioTek Synergy 2 plate reader at 570 nm, with a reference wavelength of 600 nm. The mitochondrial viability measurements of 4 culture plate wells (technical replicates) were normalized to the associated SCC NT controls and averaged per culture (biological triplicates).

### 2.4. Annexin V (AV) and 7-Aminoactinomycin D (7-AAD) Survival Assay

At harvest, adherent and floating β-cells were collected and stained with AV, which binds phosphatidylserine on the outer leaflet of the cell membrane during early apoptosis and 7-AAD, which intercalates with the DNA of dead and dying cells (BD Pharmingen Apoptosis Kit, 559763). Stained samples were analyzed with the Accuri C6 Plus Flow Cytometer, and cell population gaits were assigned using unstained and single stained controls. After selecting the singlet data events, unstained cells were designated as healthy, while AV positive, 7-AAD positive, and double-positive populations were aggregated to represent dead and dying cells (Figure S2). Identical compensation and gating were applied to all experimental samples, and the resulting quadrant percentages were analyzed. The percent healthy and the percent dying values of 3 culture plate wells (technical triplicates) were averaged per culture (biological triplicates).

### 2.5. [3H]-Thymidine Incorporation Proliferation Assay

To measure β-cell proliferation rates, treatment medium was replaced with medium containing radiolabeled [methyl-3H]-thymidine (PerkinElmer, Waltham, MA, USA, NET027E001MC), as described previously [95]. DNA was precipitated with 10% trichloroacetic acid and solubilized with 0.3 M NaOH, and incorporation was measured by liquid scintillation counting. Counts per minute were normalized to total protein measured by the bicinchoninic acid assay (BCA) (ThermoFisher Scientific, Waltham, MA, USA, 23225). These proliferation values of 4 culture plate wells (technical replicates) were normalized to the associated SCC NT controls and averaged per culture (biological triplicates).

### 2.6. Glucose-Simulated Insulin Secretion (GSIS) Assay

At harvest, β-cell and islet treatment medium was replaced with secretion assay buffer containing 2.8 mM followed by 12 mM glucose, as described previously [55,96]. TMAO treatment concentrations were maintained in the buffer during static stimulation incubations. Cells were lysed, and insulin concentration in secretion samples and lysate were measured by enzyme-linked immunosorbent assay, as described previously [97]. Insulin standards were purchased separately (Monobind Inc., Lake Forest, CA, USA, 2425-300) and used to quantify these sample concentrations. Insulin measurements were normalized by total protein measured by the BCA. These insulin values of 4 culture plate wells (technical replicates) were normalized to the associated SCC NT controls and averaged per culture (biological triplicates).

### 2.7. Scanning Transmission Electron Microscopy (STEM)

For STEM imaging, INS-1 β-cells were seeded to coverslips, and groups of three coverslips were cultured with treatments as described in Section 2.2 (biological triplicates). At harvest, cells were fixed, stained, sectioned, and imaged on a scanning electron microscope in STEM mode at the BYU Electron Microscopy Facility. Cytosol area and granule counts were manually quantified using ImageJ, as described previously [54,98,99]. The number of mature and immature insulin granules per total cytosolic area was quantified for each image. We averaged counts for three images (technical triplicates) per culture, yielding 9 total images analyzed per experimental treatment. Values were not normalized to report untransformed values.

### 2.8. Glutathione (GSH) Assay

After experimental culture, β-cells were lysed, and concentrations of GSH and glutathione disulfide (GSSG) were determined via high performance liquid chromatography, as described previously [100,101]. Concentrations were measured as S-carbosymethyl, N-dansyl derivatives using an internal standard for quantification, and values were corrected by total protein and were expressed as nmol GSH/mg protein [102]. Redox potentials (Eh) were determined via the Nernst equation, as described previously [103]. Quantification of the S-glutathionylation of proteins (Pr-SSG) was determined by the reduction of modified proteins, as described previously [104]. In short, harvested protein pellets were re-solubilized in buffer containing dithiotrheitol to remove GSH from Pr-SSG before derivatization for HPLC analysis. These measurements of 3 culture plate wells (technical replicates) were averaged per culture (biological triplicates).

### 2.9. qPCR

For qPCR, β-cells were lysed, and mRNA was harvested as previously described [105]. qPCR was performed using the Life Technologies Quant Studio 6 Detection System and Software (Thermo Fisher), using SYBR green primers and qPCR master mix (Bio-Rad, Hercules, CA, USA), as previously described [105] for Total XBP1, sXBP1, and PPIA. Relative mRNA levels for Total XBP1 and sXBP1 were calculated using the Delta CT method, with PPIA being used as a housekeeping gene. Sequences are available upon request.

### 2.10. Western Blots

For Western blots, β-cells were lysed, and blotting, transfers, and imaging (LI-COR Biotechnology) were performed as described previously [55]. Protein concentration was determined by BCA, and samples were loaded in the gel at 25 µg/lane. Blots were probed for inositol-requiring enzyme 1α (IRE1α) (Cell Signaling, Danvers, MA, USA, 3294, 1:1000), PRKR-like Endoplasmic reticulum kinase (PERK) (Cell Signaling, 3192, 1:1000), phosphor-PERK (Cell Signaling, 3179, 1:1000), ATF4 (GeneTex, Irvine, CA, USA, GTX101943, 1:1000), and X-box bind protein 1 (XBP1) (GeneTex, GTX102229, 1:1000), and Tubulin (Proteintech, Rosemont, IL, USA, 66031-1-Ig, 1:25,000) as a loading control. These expression measurements of 3 culture plate wells (technical replicates) were normalized to the associated SCC NT controls and averaged per culture (biological triplicates).

### 2.11. Statistical Analysis

Data presented in the text represent mean percent change between SCC NT and GLT NT controls. As indicated in each experimental method and figure legend, the mean of 3 to 4 technical replicates constituted the measurements of 3 to 4 predetermined biological replicates (n = 3 or 4), with error bars indicating the standard error. For INS-1 β-cell cultures, each independent culture plate, and for primary islet cultures, each independent animal, was considered a biological replicate. Statistical analyses were performed with GraphPad Prism 9, where *p* < 0.05 was considered significant, but instances of *p* < 0.1 were also reported. Statistical comparisons included the normality test using the Shapiro Wilk test, one-way ANOVA with Tukey’s post hoc test, and two-way ANOVA with Šidák’s post hoc test, as indicated in the figure legends.

## 3. Results

### 3.1. TMAO Does Not Alter GLT-Mediated Reduction of β-Cell Mass

**Figure 1:**
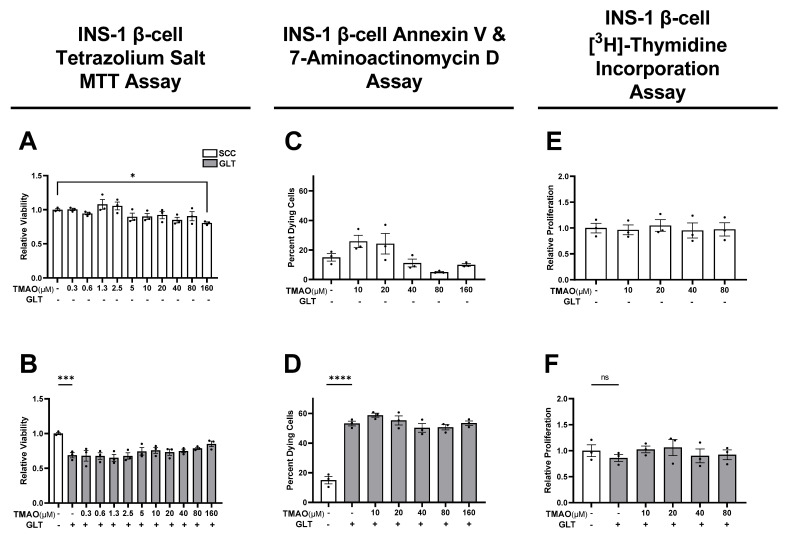
Trimethylamine N-oxide (TMAO) does not alter glucolipotoxicity (GLT)-mediated reduction of INS-1 β-cell mass. (**A**) Tetrazolium salt MTT measured mitochondrial viability of INS-1 β-cells cultured with or without TMAO for 24 h in standard cell culture (SCC), and (**B**) in GLT. (**C**) Flow cytometric measurement of dead and dying β-cell populations as measured by Annexin V and 7-aminoactinomycin D staining of β-cells cultured with or without TMAO for 24 h in SCC, and (**D**) in GLT. (**E**) β-cell proliferation measured by [3H]-thymidine incorporation of β-cells cultured with or without TMAO for 24 h in SCC, and (**F**) in GLT. (**A**,**B**,**E**,**F**) Values are normalized to SCC no treatment (NT) controls and (**C**,**D**) values reported as percent. (**E**,**F**) Proliferation data were normalized to cell protein content. All values represent the mean of biological triplicates (n = 3). Error bars indicate the standard error. * Indicates significant one-way ANOVA showing GLT and TMAO effects with *p*-values * <0.05, *** <0.001, **** <0.0001, or not significant (ns).

Since impaired functional β-cell mass is a hallmark of T2D pathogenesis, we explored the viability, survival, and proliferation rates of TMAO-treated β-cells in SCC and GLT conditions (Figure 1). Mitochondrial viability assays showed that 160 µM TMAO was cytotoxic under SCC conditions (Figure 1A), and GLT reduced mitochondrial viability by 31% (Figure 1B). Since this assay reports mitochondrial viability as a surrogate for cell mass, we investigated β-cell survival via dead and dying cellular markers AV and 7-AAD. There was minimal cell death and no significant TMAO effect in SCC studies (Figure 1C), while GLT increased the dead and dying population by 38% (Figure 1D). Finally, we measured β-cell proliferation by quantifying DNA synthesis rates where TMAO showed no effect (Figure 1E,F). While GLT did significantly reduce total protein concentration during the [^3^H]-thymidine assay, when DNA synthesis rates were corrected by these values, the comparisons were not significant (Figure S1C–E). Together, these data illustrate that while GLT reduces β-cell mass by decreasing mitochondrial viability and increasing cell death, TMAO does not alter β-cell mass.

### 3.2. TMAO Normalizes GLT-Damaged β-Cell and Islet Function

**Figure 2:**
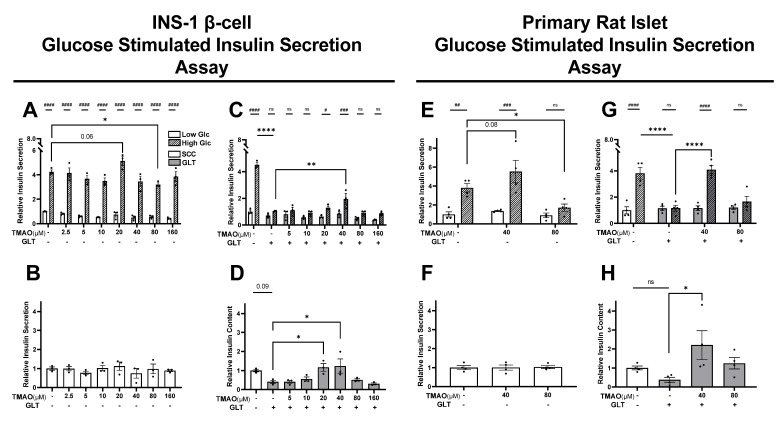
TMAO normalizes GLT-damaged INS-1 β-cells and primary rat islet function. (**A**) Insulin secretion under low (solid bars) vs. high glucose (striped bars) for INS-1 β-cells cultured with or without TMAO for 24 h in SCC condition, and (**B**) corresponding insulin content. (**C**) Insulin secretion from β-cells cultured with or without TMAO for 24 h in GLT condition, and (**D**) corresponding insulin content. (**E**) Insulin secretion from primary rat islets cultured with or without TMAO for 24 h in SCC, and (**F**) corresponding insulin content. (**G**) Insulin secretion from islets cultured with or without TMAO for 24 h in GLT, and (**H**) corresponding insulin content. All values are normalized to SCC NT controls and represent the mean of biological replicates (n = 3 or 4). (**A**,**C**,**E**,**D**) # Indicates significant paired two-way ANOVA comparing low vs. high glucose. (**A**–**H**) Error bars indicate the standard error. * Indicates significant one-way ANOVA showing GLT and TMAO effects, where *p*-values < 0.1 given, * <0.05, ** <0.01, **** <0.0001, or ns.

To investigate TMAO effects on β-cell function, we performed GSIS assays to measure the insulin secretion and production capacity of β-cells treated with TMAO under SCC and GLT conditions (Figure 2A–D). SCC only controls secreted 4.4-fold more insulin during 12 mM high glucose stimulation compared to the 2.8 mM low glucose stimulation (Figure 2A,C). This significant difference between the low and high glucose insulin secretion values indicates healthy glucose sensing, which is hindered by GLT (Figure 2C). GSIS was worsened by 80 µM TMAO in the SCC condition (Figure 2A), while 20 and especially 40 µM TMAO improved GLT-blunted GSIS (Figure 2C). To explore β-cell insulin production, we measured total insulin content (Figure 2D). TMAO did not significantly change insulin content in SCC treated cells (Figure 2B), but 20 and 40 µM TMAO in the GLT condition recovered insulin content to healthy levels (Figure 2D). These results demonstrate a beneficial phenotype, where physiological concentrations of TMAO maintained β-cell insulin secretion and content during the damaging T2D-like GLT condition.

Primary rat islet studies validate this beneficial TMAO phenotype (Figure 2E–H). Female mice are a common model in TMAO studies because their increased FMO3 expression and activity produces four-fold more TMAO than males [28]. However, because adult humans show a less pronounced sexual dimorphism in TMAO accumulation, we cultured adult female rat islets as a more relevant model [28,106,107,108]. As with β-cells, islet insulin section was inhibited by 80 µM TMAO in SCC, while insulin content was unaffected (Figure 1E,F). Acute 40 µM TMAO improved GSIS and insulin content levels in GLT cultured β-cells (Figure 2G,H). These islet data corroborated the β-cell results that 40 µM TMAO is sufficient to normalize β-cell function impeded by GLT. While GSIS function is known to be dependent on mitochondrial function, we did not observe matching TMAO effects in the mitochondrial viability of 40 µM TMAO treated GLT cultured β-cells (Figure 1B). Therefore we hypothesize that the increased insulin secretion observed here (Figure 2C,G) may be due to an increase in insulin production, as indirectly measured by the insulin content values (Figure 2D,H).

### 3.3. TMAO Recovers the Decrease in Insulin Granule Number Induced by GLT

**Figure 3:**
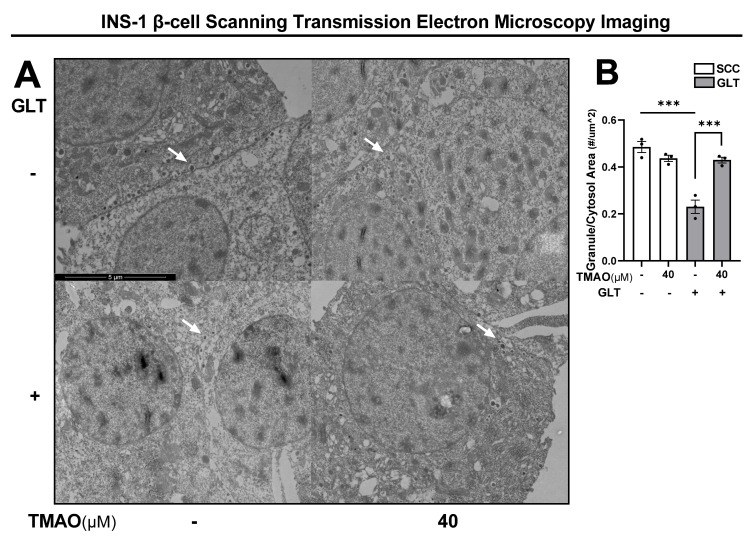
TMAO recovers the decrease in INS-1 β-cell insulin granule number induced by GLT. (**A**) Representative scanning transmission electron microscopy images of INS-1 β-cells cultured with or without 40 µM TMAO for 24 h in SCC and GLT. White arrows identify examples of insulin granules. (**B**) Quantification of insulin granules/cytosol area. All values represent the mean of biological triplicates (n = 3). Error bars indicate the standard error. * Indicates significant one-way ANOVA showing GLT and TMAO effects, where *p*-values *** <0.001.

To more directly explore the TMAO effects on insulin production, we used STEM imaging to visualize β-cell insulin granule formation (Figure 3A). GLT reduced granule number per cell by 53% (Figure 3B). While TMAO did not alter granule numbers in SCC, in the GLT condition 40 µM TMAO was sufficient to recover granule counts to healthy levels (Figure 3B). Therefore, TMAO presumably facilitates proper insulin production by maintaining mature granule formation despite deleterious GLT damage.

### 3.4. TMAO Does Not Alter GLT-Mediated Changes to GSH or Redox Potential

**Figure 4:**
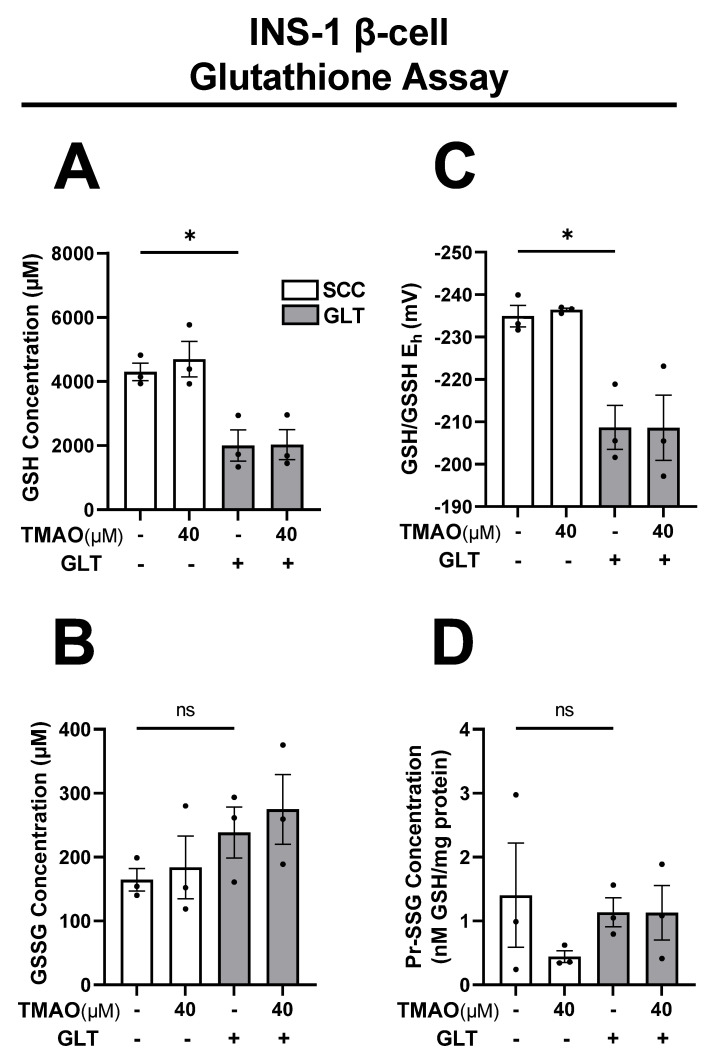
TMAO does not alter GLT-mediated changes to GSH or redox potential in INS-1 β-cells. INS-1 β-cells cultured with or without 40 µM TMAO for 24 h in SCC and GLT were measured for (**A**) Glutathione (GSH) concentration, (**B**) Glutathione disulfide (GSSG) concentration, (**C**) GSH/GSSG redox state, and (**D**) Protein S-glutathionylation (Pr-SSG) concentration. All values represent the mean of biological triplicates (n = 3). Error bars indicate the standard error. * Indicates significant one-way ANOVA showing GLT effects, where *p*-values * <0.05 or ns.

GLT is associated with increased reactive oxygen species (ROS) levels causing endoplasmic reticulum (ER) stress and mitochondrial dysfunction, which damage β-cell function [109,110,111,112,113,114,115]. β-cells are vulnerable to oxidative stress due to their limited endogenous antioxidant defense system, and reducing ROS levels is sufficient to recover healthy function [43,44,47,113,116,117,118]. Therefore, we investigated TMAO effects on the endogenous antioxidant GSH (Figure 4). GLT altered the redox potential (Figure 4C) of the β-cells by decreasing the GSH concentration by 47% (Figure 4A), without significantly affecting the GSSG (Figure 4B) or Pr-SSG concentrations (Figure 4D). It was shown that 40 µM TMAO had no protective effects against these changes and that the beneficial TMAO phenotype we observed is not linked to alleviating oxidative stress caused by GLT.

### 3.5. TMAO Normalizes GLT Mediated Changes to Unfolded Protein Response (UPR) Components

**Figure 5:**
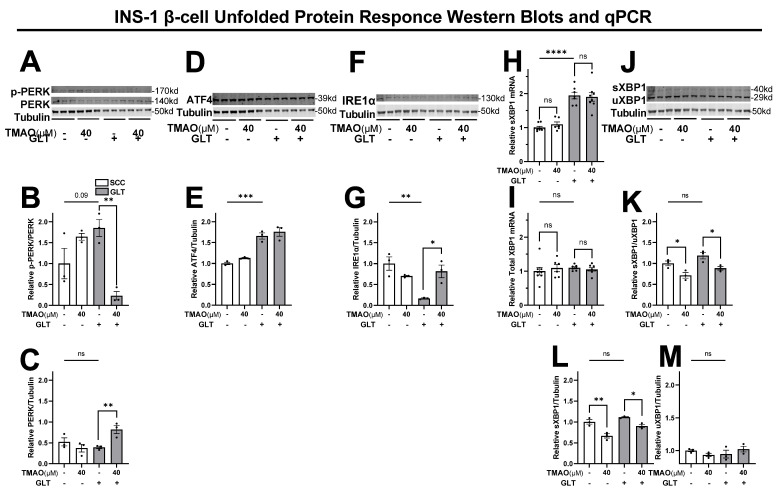
TMAO normalizes GLT-mediated reduction of the IRE1α unfolded protein response (UPR) in INS-1 β-cells. INS-1 β-cells cultured with or without 40 µM TMAO for 24 in SCC and GLT were measured for changes to UPR pathway proteins by Western blotting and mRNA by qPCR. Samples were probed and quantified for p-PERK (170kd) and PERK (140kd) (**A**–**C**), ATF4 (39kd) (**D**,**E**), IRE1α (130kd) (**F**,**G**), sXBP1 (40kd) and uXBP1 (29kd) (**J**–**M**). mRNA of sXBP1 and total XBP1 were measured by qPCR (**H**,**I**). Abbreviations: quantitative polymerase chain reaction (qPCR), phospho-protein kinase RNA-like ER kinase (p-PERK), activating transcription factor (ATF4), inositol-requiring enzyme 1α (IRE1α) (**A**,**B**), spliced and unspliced X-box bind protein 1 (sXBP1, uXBP1). All Western blot values are normalized to the loading control tubulin and represent the mean of biological triplicates (n = 3). qPCR data represent the mean of 6 to 8 replicates (n = 6–8). Error bars indicate the standard error. * Indicates significant one-way ANOVA showing GLT and TMAO effects, where *p*-values <0.1 given, * <0.05, ** <0.01, *** <0.001, **** <0.0001, or ns.

Insulin production begins with translation of the insulin mRNA at the rough ER before it is matured and packaged into granules. Therefore, ER stress triggered by unfolded protein accumulation during glucolipotoxicity is highly relevant to T2D β-cell dysfunction. We investigated TMAO effects on two arms of the UPR pathway (Figure 5). GLT slightly induced p-PERK expression, which resulted in a 77% reduction by 40 µM TMAO, indicating that TMAO reversed the GLT-mediated ER stress (Figure 5A,B). Interestingly, total PERK levels were significantly upregulated by TMAO treatment under GLT conditions (Figure 5A,C). While TMAO reduced p-PERK levels under GLT, it did not affect its downstream target, ATF4, which was elevated under GLT conditions (Figure 5D,E). Since ATF4 signals apoptosis, this non-significant result corresponds with the previous β-cell survival data (Figure 1D). In the second arm of the UPR, GLT reduced IRE1α expression by 83%, which was normalized by TMAO (Figure 5F,G). IRE1α splices the XBP1 mRNA to yield two populations of XBP1 proteins. Measurements of total and spliced XBP1 (sXBP1) demonstrated that GLT resulted in similar induction of sXBP1 mRNA (Figure 5H,I). Western blotting demonstrated that under SCC and GLT conditions, TMAO reduced spliced XBP1 relative to the unspliced population (uXBP1) and relative to the tubulin control (Figure 5J–L). However, since changes in this protein were not observed in the GLT independent of TMAO, these minimal changes may not be physiologically meaningful. These data demonstrate that GLT induces ER stress and diminishes the IRE1α UPR, which 40 µM TMAO recovers to healthy levels. Therefore, taken together, our results suggest that 40 µM TMAO is sufficient to normalize GLT-damaged insulin secretion and production by maintaining granule formation and recovering the IRE1α UPR in INS-1 β-cells.

## 4. Discussion

This study is the first to demonstrate physiologically relevant TMAO effects on pancreatic β-cells under healthy and T2D-like conditions (Graphical Abstract). Our data demonstrate that TMAO recues the impaired insulin content, insulin granule levels, and ultimately the insulin secretion, in part by modulating UPR proteins to presumably enhance proper insulin protein folding. To mimic the serum TMAO levels of healthy and chronic disease patients, we cultured INS-1 β-cells and primary rat islets in the presence of 0.3 to 160 µM TMAO concentrations [16,20,67,68,69,70,71,72,73,74]. Serum TMAO accumulates after a single meal rich in precursor nutrients and remains elevated for the duration of the diet [13,16,87,90,91]. Despite variability among diets and patients, serum TMAO levels are typically low in healthy subjects, ranging from 1 to 3 µM, which increases to 15 µM with old age [8,16,67,87,88,89]. The onset of Western-diet-driven chronic diseases elevates TMAO to concentrations from 7 and 170 µM, with kidney disease patients showing the highest levels and T2D or CVD patients showing levels at or above 20 µM [5,25,68,70,72,74,75,79,80,81,82,83,84,85,86]. We observed significant TMAO effects within this concentration range in the SCC and GLT cultures. The most physiologically relevant treatment combinations of this study include the lower TMAO concentrations in SCC and the higher TMAO concentrations in GLT conditions. The higher TMAO levels in SCC and the lower levels in GLT conditions represent in vitro study controls. Therefore, while we found that β-cells cultured with supraphysiological concentrations of 80 and 160 µM TMAO in SCC had impaired mitochondrial viability and GSIS (Figure 1A and Figure 2A,E), these results are less physiologically relevant. Conversely, the beneficial TMAO phenotype we observed in GLT cultured INS-1 β-cells and primary rat islets with 40 µM TMAO demonstrated normalized insulin secretion and content (Figure 2C,D,G,H), insulin granule density (Figure 3), and upregulated IRE1α UPR (Figure 5) relevant to the diet-induced T2D β-cell physiology (Graphical Abstract). Future studies on primary human islets and in vivo models may further validate the clinical relevance of these results.

The GLT condition models hyperglycemia and hyperlipidemia, which drive β-cell damage indicative of diet-induced T2D [51]. Hyperglycemia, or its model glucotoxicity, is a hallmark of early- and late-stage T2D pathogenesis [51,52,119]. Hyperlipidemia accompanies obesity and T2D and is modeled by cultures high in free fatty acids, especially palmitate, as previously studied by the authors [63,64,65,66,120]. Because hyperglycemia precedes hyperlipidemia, the GLT condition replicates physiologically relevant synergistic effects [63]. In β-cells, ROS easily overcomes the limited endogenous antioxidant defense system and stunts functional β-cell mass (Figure 1, Figure 2 and Figure 3) [42,43,44,118,121]. Our data support this framework because GLT decreased the GSH concentration and redox potential; however, TMAO showed no effect on this oxidative mechanism (Figure 4A,C).The beneficial TMAO phenotype we observed on β-cell function corresponds with increased IRE1α and decreased P-PERK protein levels during GLT-induced ER stress. β-cell function begins with insulin production via translation and folding at the ER, followed by maturation into secretory granules [41,45,46,122,123,124,125,126,127,128,129,130,131]. Therefore, ER stress and accumulation of unfolded proteins at the ER plays a major role in T2D β-cell damage [115,132,133,134]. Indeed, human, animal, and in vitro T2D studies show elevated levels of ER stress and demonstrated that reducing ER stress can recover healthy β-cell function [135,136,137]. The various arms of the UPR can maintain or modify ER function in response to ER stress or induce apoptosis during severe conditions [133,138,139,140]. The p-PERK and IRE1α arms are activated by accumulated unfolded proteins and are specifically linked with T2D β-cell dysfunction modeled by GLT [134,138,139,141]. The p-PERK arm limits protein translation rates to reduce the load of proteins in the ER and can trigger apoptosis via its downstream target ATF4 [139,140]. Our data (Figure 1D and Figure 5A–E) support this framework. We found that 40 µM TMAO reduced p-PERK but not ATF4 expression (Figure 5A,B,E,G) nor survival rates (Figure 2D). While our study does not identify the whole p-PERK arm as part of the beneficial TMAO mechanism, others studies show closer associations between PERK and TMAO, albeit at supraphysiological concentrations or in other cell types [6,121]. Instead, we propose that TMAO shifts the UPR toward the IRE1α arm, which increases the ER protein folding capacity [134,138,139,141]. Low IRE1α expression, as shown in the GLT condition (Figure 5F,G), specifically increases β-cell susceptibility to ER stress in the context of T2D [133,138,142]. Indeed, IRE1α-deficient β-cells and mice demonstrate hyperglycemia and hypoinsulinemia and impaired insulin production, which recapitulates the GLT effect on cultured β-cells and islets, which we observed (Figure 2) [133,143,144,145,146]. Furthermore, these studies link the IRE1α pathway with adaptive autophagy mechanisms that enhance islet function similar to the beneficial TMAO phenotype we observed [133,143,144,145]. TMAO is also defined as a direct protein folding chaperone, independent of its action on protein expression [35,147,148,149,150,151,152,153,154,155]. Molecular dynamics studies reveal that TMAO acts as a surfactant between the folding protein and the aqueous ER environment to selectively stabilize collapsing proteins [29,31,156,157,158]. Therefore, our results add to others that identify beneficial molecular TMAO effects on metabolic tissue function. We conclude that 40 µM TMAO normalizes β-cell function by activating the IRE1α UPR to maintain insulin production and granule formation despite GLT-induced ER stress.

While we demonstrate beneficial TMAO effects on β-cell function, some studies using other tissue types counter this conclusion [6,159]. Human and animal in vivo studies show improved hyperglycemia and hyperlipidemia [11,28,160,161]. While the glucose tolerance test (GTT) results of these studies may point toward deleterious TMAO effects on β-cells, this method did not directly quantify β-cell function through mathematical modeling [162]. Instead, these studies correlated the blood glucose level changes with altered insulin responsivity in the target tissue function [163,164,165,166,167,168,169]. While these studies generally conclude deleterious TMAO effects in various metabolic tissues, none have investigated TMAO effects directly at the β-cell level, which is critical to T2D pathogenesis [6,21,24,25,26,27,28].

Although some studies contradict our findings as discussed above, others corroborate that TMAO protects against T2D conditions [30,110,170,171]. In vitro studies of other metabolic tissues demonstrate that reduced TMAO levels exacerbate cellular damage, while elevated TMAO levels recover healthy phenotypes [171,172,173,174]. A study on TMAO-treated diabetic mice showed fewer neuropathy symptoms and identified TMAO as a protective protein folding chaperone [175]. Like β-cells, the neuronal secretory function relies on proper ER protein folding and secretion mechanisms. Together, these studies begin to highlight the beneficial roles of TMAO across various metabolic tissues.

Although this study is the first to identify the TMAO effects directly at the β-cell, another study agrees that TMAO benefits glucose tolerance in HFD-fed T2D mice [170]. This showed that the HFD-fed mice with experimentally elevated serum TMAO levels performed better on a GTT compared to control mice. A GSIS experiment on isolated islets cultured with TMAO in this study also demonstrated beneficial TMAO effects on insulin secretion [170]. Although this study did not report serum TMAO levels, this in vivo study closely approximated the beneficial effects we observed in T2D β-cells and rat islets treated with 40 µM TMAO [170].

## 5. Conclusions

We conclude that acute 40. µM TMAO treatment maintains healthy β-cell function during the GLT-mediated T2D condition by normalizing insulin granule formation, increasing the IRE1α, and decreasing the p-PERK UPR. Further evaluation of TMAO on primary human islets or via in vivo studies will provide clearer insight into the protective effects we observed here. Since the landmark study identifying TMAO as a biomarker of CVD only 10 years ago, a general role for TMAO across chronic diseases has been debated, and some suggest that TMAO effects in the context of T2D may differ from those typically supported by CVD research [8,9,23,25,30,176,177,178,179,180]. Indeed, it is proposed that TMAO may initiate protective or compensatory cellular responses to diet-mediated metabolic diseases [23,181,182]. Our data demonstrate crucial findings to support such a context-dependent role for TMAO and suggest that TMAO acts as a signal from the diet-microbiome interaction to metabolic tissues to trigger adaptive cellular responses to the overnutrition-driven cellular stress. We propose that at the β-cell, acute TMAO initiates protective effects that may mitigate diet-mediated T2D pathogenesis.

## Supplementary Materials

The following are available online at https://www.mdpi.com/article/10.3390/biom11121892/s1, Figure S1. Glucolipotoxicity (GLT) component effects on INS-1 β-cell glucose stimulated insulin secretion (GSIS) and prolifer-ation. Figure S2. Flow cytometry gating criteria for INS-1 β-cell survival analysis.
