# Supplementary material for: Gut Metabolite Trimethylamine N-Oxide Protects INS-1 β-Cell and Rat Islet Function under Diabetic Glucolipotoxic Conditions
Source: Biomolecules. 2021 Dec 17;11(12):1892. doi: 10.3390/biom11121892 (PMC8699500; doi:10.3390/biom11121892)
Supplement: Supplementary file 1 [file biomolecules-11-01892-s001.zip › biomolecules-1468398-supplementary.pdf]

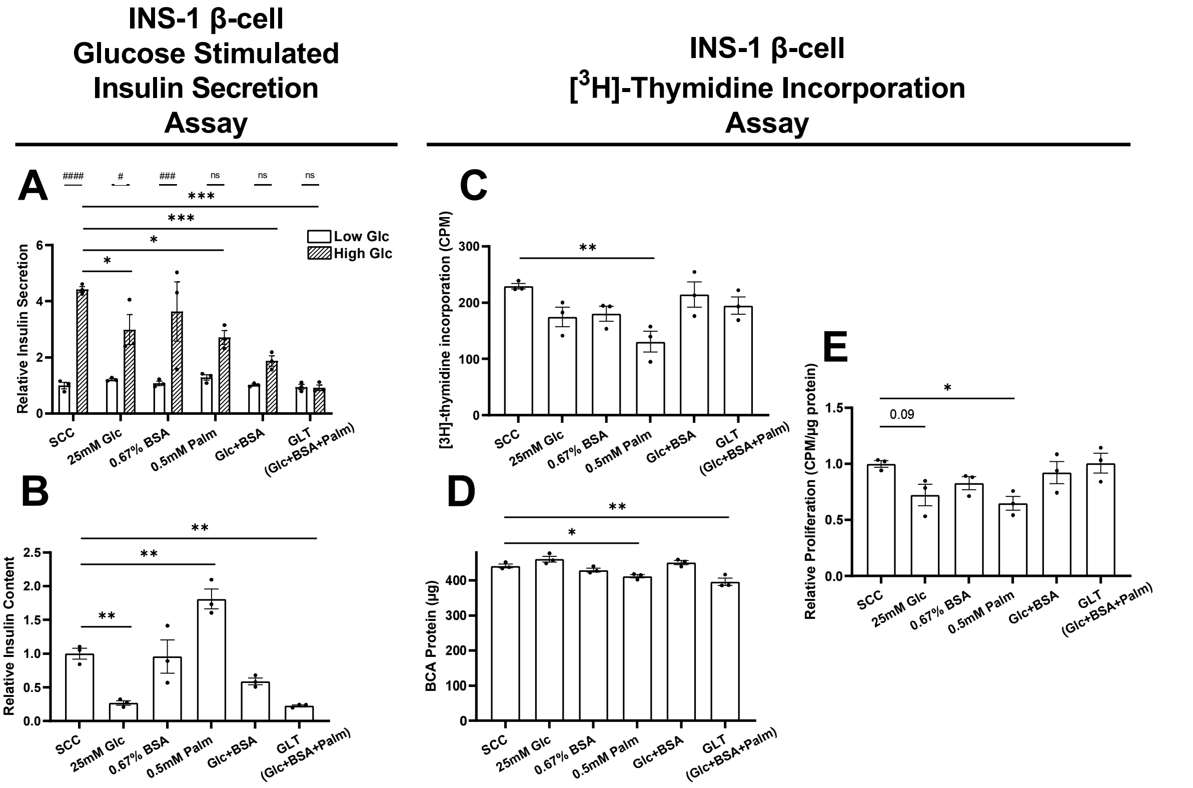

Supplemental Figure S1. Glucolipotoxicity (GLT) component effects on INS-1 β-cell glucose stimulated insulin secretion (GSIS) and proliferation. (A) Insulin secretion results for INS-1 β-cells in the standard culture condition (SCC), the complete GLT condition, or media with individual GLT components or partial combinations for 24 hours. (B) Insulin content results for β-cells in A. (C) [3H]-thymidine incorporation counts per minute (CPM) showing DNA synthesis rates for β-cells cultured as in A. (D) Bicinchoninic acid assay (BCA) total protein results for β-cells cultured as in A. (E) Corrected DNA synthesis rates for β-cells cultured as in A. Abbreviations: Glucose (Glc), bovine serum albumin (BSA), palmitate (Palm). A,B,E values are normalized to SCC controls and represent the mean of biological triplicates (n=3). C,D values are untransformed. # Indicate significant paired two-way ANOVA comparing low vs high glucose. * Indicate significant one-way ANOVA compared to SCC controls. p-values <0.1 given, *<0.05, **<0.01, ***<0.001, or ****<0.0001.

INS-1 β-cell
Annexin V & 7-Aminoactinomycin D
Flow Cytometry Assay

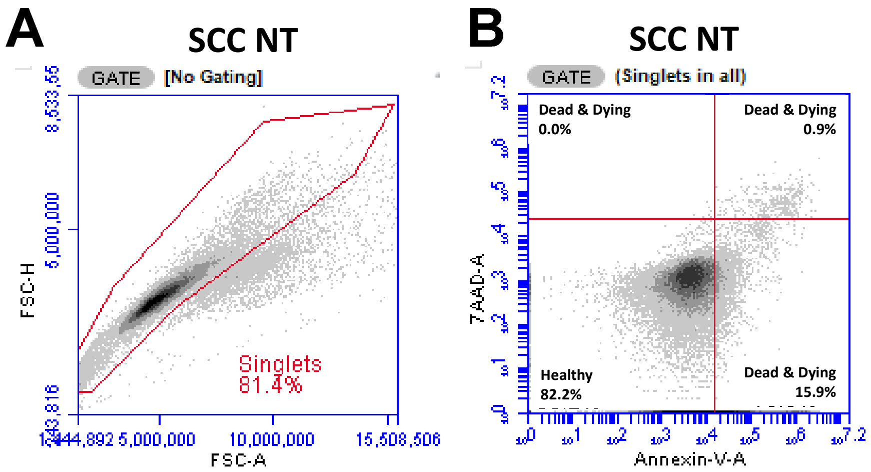

Supplemental Figure S2. Flow cytometry gating criteria for INS-1 β-cell survival analysis.

(A,B) Density plots generated by the Accuri C6 Plus Flow Cytometer for a representative SCC no treatment (NT) control INS-11 β-cell sample after staining with Annexin V (AV) and 7-Aminoactinomycin D (7-AAD) for survival analysis. (A) Singlets were gated in red and included in (B) where quadrants were assigned to define the double negative population in the lower left (Healthy) and the remaining quadrants were aggregated (Dead & Dying). Abbreviations: Forward scatter height (FSC-H), forward scatter area (FSC-A).
